# Informal and Formal Supports for Former Child Soldiers in Northern Uganda

**DOI:** 10.1100/2012/825028

**Published:** 2012-12-31

**Authors:** Sofie Vindevogel, Michael Wessells, Maarten De Schryver, Eric Broekaert, Ilse Derluyn

**Affiliations:** ^1^Department of Orthopedagogics, Centre for Children in Vulnerable Situations, Ghent University, H. Dunantlaan 2, 9000 Gent, Belgium; ^2^Heilbrunn Department of Population and Family Health, Program on Forced Migration and Health, Columbia University, 60 Haven Avenue, B-4, Suite 432, New York, NY 10032, USA; ^3^Department of Experimental, Clinical, and Health Psychology, Ghent University, H. Dunantlaan 2, 9000 Gent, Belgium

## Abstract

This study aimed to evaluate the potential contribution of informal community initiatives and formal interventions in support of former child soldiers' resilience in the wake of armed conflict. Using a cross-sectional survey design, a stratified random sample of 330 formerly recruited and 677 nonrecruited young people was consulted about their perspective on desirable support for former child soldiers provided by close support figures, communities, humanitarian organizations, and governments. Data analysis occurred by conducting qualitative thematic analysis and statistical chi-square analysis to explore clusters, similarities, and variations in reported support across the different “agents,” hereby comparing the perspectives of formerly recruited and non-recruited participants. The results indicated that formerly recruited and non-recruited participants had comparable perspectives that call for the contribution of various informal and formal support systems to former child soldiers' human capacities and the communal sociocultural fabric of war-affected societies. This highlights the importance of community-based, collective, and comprehensive support of formerly recruited young people and their surroundings in the aftermath of armed conflict.

## 1. Introduction

Contemporary warfare increasingly inflicts military strategies on civilians and sometimes particularly victimizes children (minor 18s) [1]. Among the more notorious and devastating war strategies is the recruitment of children by armed groups and forces. It is estimated that currently about a quarter of a million children are conscripted and militarily engaged in armed conflicts worldwide [2]. Such child soldiering experiences typically involve persistent and intense exposure to war-related adversity, which constitutes a severe threat to the mental health of these children [3, 4]. As a consequence, substantial psychological distress has consistently been assessed in former child soldiers [5–8]. Additionally, child soldiering also inflicts harm on the physical, social, educational, and economic aspects of their lives and therefore creates multiple challenges [9–11]. This potentially degrades former child soldiers' capacities upon return from the armed group or armed force and may lead to considerable loss of “human capital,” which refers to the resources endowed to individuals [12]. However, the impact of child soldiering reaches far beyond the individual level. Targeting civilians as a war-strategy profoundly disrupts familial networks, social cohesion, civic services, and therefore destabilizes the entire social ecology of affected communities. The “social ecology” refers to the social context in which individuals develop and that offers the social capital that they can use in responding to encountered challenges [12]. War strategies targeting civilians also erode traditional practices, mores and values and defy human rights in the affected community, thereby rupturing the “cultural capital,” that is, the resources emanating from cultural and moral frameworks [12–16]. These multiple and intersecting ways in which child soldiering impinges on the psychosocial well-being of formerly recruited young people bring along implications for their transition from military to civilian life and for desirable support in the aftermath of the child soldiering episode [17–20]. 

These implications have been incorporated into the Psychosocial Working Group (PWG)'s conceptual framework for psychosocial intervention in complex emergencies [12]. This framework forms an integration of resource-based approaches (e.g., conservation of resources theory [21, 22]) and social ecological approaches to child development (e.g., ecological systems theory [23]). The PWG theoretical framework delineates how people and communities at large deal with potential or actual loss of human, social and cultural capital in complex emergencies. When facing loss of such resources, people strive to maximize gain and to minimize loss in order to obtain and preserve resources that help in dealing with chronic and acute challenges [21, 22]. As such, formerly recruited young people may seek to reactively repair the damage caused by child soldiering and to proactively protect their resources against the possible cascading demands that are associated with its aftermath. Through the use of such resources, many former child soldiers are able to maintain or regain well-being despite the unpromising circumstances, in a process which is termed “resilience” [24]. Hereto, they actively engage to gain support for the extant resources and create new, supplementary resources [25]. Additionally, when confronted with an adversity such as armed conflict, affected communities tend to strengthen their informal support systems and to actively engage in self-help processes to address the challenges in their situation [12, 26]. In this process, a myriad of resources is employed and socially exchanged to counter the inflicted harm and to proactively bolster one another's well-being [21, 22]. Thus, agents in the environment are mediating the individual's access to supplementary resources in the collective resource pool. This points to the important intersections between individual and collective processes in response to the potential or actual demands associated with child soldiering and war at large. These indigenous sources and processes of support that communities use to enable well-being of their members is referred to as “community resilience” [12, 27, 28]. It is expected that by virtue of community resilience and the presence of these indigenous resources, the majority of former child soldiers is able to maintain or regain well-being [28].

All this raises questions concerning the role that formal support systems, such as governmental agencies and non-governmental organizations, must fulfill and the necessity and complementarity of their services in conjunction with those already provided by informal support systems. The initiation of formal support in (post-)conflict settings often follows the assumption that the informal support systems have insufficient resources or engage insufficiently in resource exchange processes to deal with the formidable harm inflicted on them [12, 20]. By doing so, programmatic responses risk disregarding the remarkable resourcefulness and resilience of war-affected individuals and their communities [12, 20]. While there is a consensus that the availability of indigenous resources and supportive responses is far from antithetical to the need for professional interventions [28], different perspectives exist on the desirable focus areas, methods of operation and position to take when intervening in war-affected areas. This study aims at addressing this issue through consulting former child soldiers' perspective on what different agents could ideally do to support them in the aftermath of their child soldiering experience. It is expected that what is proposed as desirable support covers a broad range of domains and is accounted for by different “agents” that are either informally or formally involved. Since resilience is largely dependent on the response of the environment and the extent to which agents in this environment invest and exchange resources [12, 29], it is also important to know whether the environment acknowledges and endorses the agents' supportive role towards former child soldiers. As an initial attempt to explore the views of close support figures, this study examined the perspectives of former child soldiers' age mates with regard to what different agents should do to support formerly recruited young people. Since former child soldiers frequently were found to be stigmatized [5, 30–32], the hypothesis seemed plausible that their age mates tended to think that formerly recruited youths themselves are to blame for their situation, which therefore they should resolve on their own with little support from environmental agents. 

## 2. Methods

This study is part of a larger mixed-method research project conducted between October and December 2010 in the Lira district of northern Uganda. This area is currently in transition after two decades of a complex armed conflict in which the Lord's Resistance Army (LRA) forcibly recruited thousands of minors as child soldiers [5]. The aim of the research was to assess challenges and resources in the transition of formerly recruited young people, whereby this study specifically aimed to enhance the understanding of how different agents can contribute to this transition and eventually to well-being in the wake of child soldiering. Hereto, the study took a contextually grounded, participatory approach that was approved by the Ethical Committee of the Faculty of Psychology and Educational Sciences of Ghent University. 

### 2.1. Participants

To create a stratified random sample, the District Education Office's overview of schools in Lira district was used to select six secondary schools and for each school two adjacent villages in urban, periurban, and rural areas. The age range of 12–25 years was determined to include youth that were at the time of the LRA insurgency most likely to be among the young people that the LRA targeted for recruitment. In the villages, the out-of-school youth in this age bracket was invited to participate. In the schools, the students of classes Senior 2 and 3 of the O-level were considered to be the best age-match, given that in the lower level a diversity of ages was represented and that the higher level showed a considerable frequency of drop-outs. This resulted in a sample of 1008 Ugandan youths, of whom about a third had formerly been recruited by the LRA (one participant did not disclose his status). 

### 2.2. Procedure

In cooperation with community leaders and school principals, a plenary meeting was organized in each village and school to disperse information necessary to make an informed decision on participating in this study. This information mainly included the purpose and procedure of the study, the possibilities and consequences of refusal or withdrawal from the study, and the availability of psychosocial support during or subsequently to the study. The written informed assent or consent was obtained from all participants. Collecting consent of legal guardians of minors was hindered by them living separately and often far apart. The participants did not receive any compensation for their participation in this study. 

A cross-sectional survey questionnaire with mainly open-ended questions was administered. This questionnaire firstly consisted of sociodemographics of the participants, including age, gender, district and location of residence, occupation, religion, household composition, and former child soldiering experiences. Secondly, it contained open-ended questions on what different agents could do to support formerly recruited young people in their transition from military to civilian life. The questions were carefully designed by the bicultural research team to ensure inclusion of the most relevant informal and formal agents (i.e., themselves, family, friends, community, organizations, government) and ease of understanding (e.g., What can they themselves do? What can their family do?). “Family” referred to the nuclear and extended family members; “friends” consisted of intimate friends, age mates and classmates; “community” referred to the people who are linked by social ties and the geographical location, including neighbors, social groups and local cultural, religious and political leaders; “organizations” included charitable, non-governmental and United Nations agencies; and “government” referred to national and international government bodies. Rules were made concerning how to communicate and translate this additional information, which was orally disseminated to the participants. 

The in-school participants and out-of-school participants with sufficient literacy skills individually administered a written version of the questionnaire in English (the official language of education), while the researcher and a trained bilingual research assistant remained available. For out-of-school participants with limited literacy skills, the questionnaire was in interview format administered orally by the researcher and simultaneously translated on-site into Lango (the native language of the region) by trained bilingual research assistants. These interviews took place individually in a confidential setting. 

### 2.3. Data Analysis

The answers were analyzed and divided into meaning units, whereby those that were conceptually identical were merged and each unique meaning unit received a different numerical code. This procedure resulted in composite lists of the reported unique items per agent. The analysis of these items was based on the Psychosocial Working Group (PWG)'s conceptual framework for psychosocial intervention in complex emergencies, which incorporates an integration of the original conceptual framework discerning the main domains of resources (i.e., human capacities, social ecology, culture and values) [12] and empirical elaboration of this framework defining key subdomains of resources in northern Uganda [33]. The thematic analysis was done by two blinded researchers to minimize errors in categorization. Using the software application for qualitative data-analysis Nvivo, the items were thematically analyzed and categorized according to the conceptual framework. Subsequently, cluster analysis by coding similarity was performed to visualize patterns in coded items across the agents, in order to determine the similarity of item allocation over the different agents. Jaccard similarity coefficient was calculated. Further statistical analyses were conducted in SPSS 20. Descriptive statistics were used to represent the sociodemographic characteristics of the sample and the allocation of the categorized items over the resource (sub-)domains for each agent. Chi square analysis of the data allowed to explore similarities and variations in reported resources across the different domains and agents, comparing between formerly recruited and non-recruited participants. The significance level was set at 0.01, to reduce the chance of Type I-error but still allow exploratory testing. 

## 3. Results

The subsample of 330 formerly recruited participants comprised 201 (60.91%) males and 129 (39.09%) females, with an average age of 17.04 (*sd*⁡ = 2.31, range = 12–25) years. The median duration of their recruitment in the LRA was 348.50 (*m* = 564.79, *sd*⁡ = 752.74, range = 1–6570) days. The greater part of them had escaped (*n* = 242, 74.46%) on average 5.57 (*sd*⁡ = 1.88, range = 1–10) years ago. A majority of 225 (68.2%) participants originated from Lira district, others resided here for familial, economic, or educational reasons. Most of them lived in a rural (*n* = 155, 47.26%) or periurban (*n* = 118, 35.98%) village, while the minority lived in town (*n* = 37, 11.28%) or in a camp (*n* = 18, 5.49%). Most participants were attending school (*n* = 235, 71.21%). Of the out-of-school participants, the greater part engaged in farming activities (*n* = 46, 48.42%) or declared to have no occupation (*n* = 33, 34.74%). The subsample of 677 non-recruited participants consisted of 346 (51.18%) males and 331 (48.89%) females, with the average age of 16.54 (*sd*⁡ = 1.91, range = 12–24) years. They either lived in the same village or attended the same class as the formerly recruited participants in this study. 

**Figure 1:**
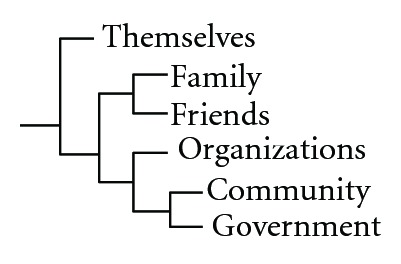
Agents clustered by coding similarity.

The cluster dendogram (Figure 1) shows a split between the items of formerly recruited individuals and the items of other agents. Among the other agents, the items of friends and family were closest related and so were those of the government and community (*J* = 1.00). The items allocated to organizations were situated in between the items of family and friends on the one hand and the items of community and government on the other hand. However, the similarity metric shows that there was high similarity between the sample sets of all agents (range = 0.80–1.00). 

**Figure 2:**
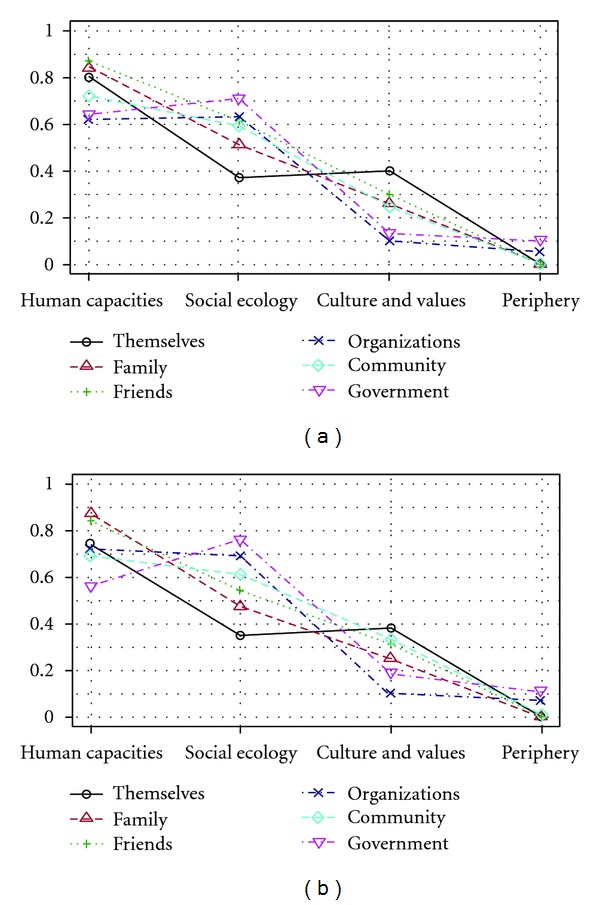
Proportion of formerly recruited (a) and non-recruited (b) youth's answers per resource domain and agent.

Table 1 and Figure 2 represent the allocation of support items per agent and per resource domain. Most items reported by formerly recruited participants pertained to support for “human capacities”, in which “knowledge and skills”, “livelihood”, and “mental health” resources were most prevailing. The reported number of items pertaining to “human capacities” was quite high for all agents, but the highest number was reported for family and friends. Another large number of items referred to support for the “social ecology”, including “social support”, “social services and infrastructure”, and “social connectedness” as the most common subdomains. Support to the “social ecology” was mostly assigned to the government, organizations and the community and the least to formerly recruited young people. The third resource domain consisted of “culture and values”, in which, respectively, “human rights”, “religious values”, and “cultural practices” were to be supported mainly by formerly recruited youths, their families and friends. The least frequently occurring items were reported for “political” and “economic” resources in the “periphery”, which were mainly assigned to governments and organizations. 

This table and figure also show that formerly recruited adolescents were primarily recommended to strengthen their own “human capacity”, by developing their “knowledge and skills”, adhering to “religious beliefs”, applying “mental health” strategies, and strengthening their “livelihood”. To a lesser extent, they were also expected to contribute to the “culture and values” and the “social ecology” of their environment. The table and figure further seem to indicate that support expected from families, friends and communities diminished steadily from resources in “human capacities” to the “periphery”. Families' largest contribution lies in the support of “human capacities”—specifically their children's “knowledge and skills”, “livelihood”, and “physical health”—and in the provision and promotion of “social support”. Friends were mainly expected to contribute to “human capacities” by supporting the “knowledge and skills” and “mental health” of their friends, and also to the “social ecology” by facilitating their “social connectedness” and delivering “social support”. The community's assignments were equally divided over the three core domains, and were more specifically oriented to support the “knowledge and skills” and “livelihood” of formerly recruited young people, as well as “social support” and “human rights” issues in the community. The support functions assigned to organizations and the government mainly pertained to the “human capacities” and “social ecology” domains, but then diminished markedly for “culture and values” and the “periphery”. Organizations were reported to make the largest contributions to “human capacities” by supporting the “livelihood”, “mental health”, and “knowledge and skills” of formerly recruited young people, and also to the “social ecology” by initiating “social services and infrastructure' and promoting “social support”. The government's responsibilities were mostly defined in relation to the “social ecology”, including the initiation of “social services and infrastructure” as well as “law and order”, and in relation to the “human capacities”, mainly the “knowledge and skills” and “livelihood” of formerly recruited youth.  Table 2 gives an illustration of the most frequently reported items for all agents in each resource domain.

Table 1 and Figure 2 further depict the comparison between formerly recruited and non-recruited youth, showing seemingly similar patterns for both comparison groups. A few significant differences appeared. Firstly, families' support to “mental health” resources was esteemed higher by non-recruited participants. Secondly, non-recruited participants reported less support from friends for “human rights” resources. Thirdly, this group reported a lower contribution of organizations to “human capacities” in general and to “social support” resources. Fourthly, communities were less supposed to support “culture and values”, and particularly “cultural practices”. Lastly, they expected governments to deliver more support to formerly recruited youth's “livelihood”, but less support to “social services and infrastructure” and to “human rights” issues.

## 4. Discussion

This study examined formerly recruited young people's perspectives on the potential contributions that diverse informal and formal support systems can make to their well-being in the wake of child soldiering. The results revealed that formerly recruited young people call for support on a variety of resource domains to which both informal and formal agents can make significant contributions. Required supports seemingly diminished from resources endowed to individuals to resources in the periphery. A plausible explanation is that those challenges and resources that are most directly related to one's well-being are often considered salient and therefore are more reported [21, 22]. Hence, the largest part of the recommendations refer to support for human capacities and more particularly for the former child soldiers' knowledge and skills, livelihood and mental health. When interpreting these most recommended types of support against the backdrop of the most pertinent challenges identified in our previous study [31], interesting parallels appear between the urge for support in “knowledge and skills” and “livelihood” resources to meet “training and skills-related” and “economic” challenges, and between support for “mental health” resources to meet “emotional” challenges. In the wake of armed conflict, formerly recruited young people may particularly face challenges related to the mental health consequences of their augmented exposure to war-related adversity and to the educational and economic impact of child soldiering [5, 7, 34]. This might explain their high demand for support in these domains and shows that there is a considerable need for specific individualized support to strengthen young people whose human capital has been threatened or affected and who consequently may experience substantial distress in the aftermath of child soldiering [1]. According to the participants of this study, such support can largely be provided by support figures among their kith and kin, but to a considerable extent also by the community-based, humanitarian, and governmental support structures. 

In addition, there is a great deal of recommendations that represent nonspecific and communal support, given that these pertain to the social and cultural fabric. The need for support of the social ecology might be explained by the fact that the impact of war is in part influenced by the extent to which social networks, public facilities, and customary practices are affected and hence limited in their supportive capacities [1, 19]. To offset the loss of social capital, social connectedness may reduce alienation and install a sense of belonging in the aftermath of child soldiering [35, 36]. Further, social support has consistently been associated with better psychosocial well-being and stronger resilience of formerly recruited youth, for it strengthens people's capacities to deal with challenges [37, 38]. Social services and infrastructure related to education, healthcare, and development among others are indispensable for human welfare. It should be noted that the limited reports with regard to safety and law and order may have been biased by the current, relatively stable and peaceful post-conflict status of northern Uganda, whereas this in the midst of conflicts is rather a primary concern and important duty to protect young people's well-being [39]. All this shows that support in the aftermath of war should work on the reconstruction of the social fabric and on the development of social capacities to support members who are in need of particular support [1, 40]. According to the participants, such support can in the first place be provided by formal support structures such as governments and organizations, but also considerably by communities, friends, and families who are at the heart of the social environment.

Support for culture and values was less reported, though still requires considerable attention from informal and formal agents. Support in this domain should mainly be oriented to human rights issues and cultural practices. During and following warfare, grave abuses may occur that defy basic human rights, and formerly recruited young people may in particular experience subjugation and discrimination, which possibly explains their need for support in this area [12]. War also often erodes the culture that unites people and constructs a shared identity, and that forms the framework for cultural-specific manifestations of challenges and responses to it [1, 19]. Support for cultural practices and values therefore is important to reinstall communal life and stimulate cooperative, indigenous responses to encountered challenges. Informal support systems fulfill an important role in reconnecting formerly recruited youth to contextually-appropriate ways of meaning-making and living, grounded in cultural, ideological, and spiritual frameworks [19]. Formal support systems should in their efforts build upon these informal and culturally grounded approaches [1, 19, 20, 40]. 

Influences of the broader context are still relevant to the formerly recruited youth, albeit apparently to a lesser extent. These peripheral factors are mainly considered to be an issue of the government that bears a duty with regard to the economic and political climate of the country. The latter should be supported by organizations [41]. The emphasis on the three core domains of resources confirms the importance of support that covers the broad range of human, social and cultural capital. It also indicates that former child soldiers' trajectories to resilience are ideally scaffolded by human, social, and cultural resources [12, 19, 20].

In addition, the study explored similarities and differences between the perception of formerly recruited and non-recruited participants concerning the contribution that various informal and formal agents can make to these resource domains. The aim of this comparison was to obtain a preliminary insight into whether former child soldiers' perspectives are endorsed by agents in their environment. The results showed some significant differences that suggest that the participants who were recruited situate more supports outside themselves and their families than is acknowledged or supported by their non-recruited age mates. Nonetheless, the overall distribution patterns of resources showed that formerly recruited and non-recruited young people generally shared the same perspective on support for former child soldiers. This reveals that the age mates acknowledge the important supportive role to be played by environmental agents, and that they are willing to invest and exchange their own and communal resources in support of formerly recruited young people. However, formerly recruited young people themselves are also expected to not only invest in their own capacities, but to make a contribution to their social ecology and to the culture and values of their community. Such investment in the socio-cultural fabric may be warranted when they aim to obtain access to the rich reservoir of communal resources in their environment, that can help to deal with loss brought forth by child soldiering and to offset ensuing challenges that may occur in the aftermath [25]. Various support figures and systems can act as mediator to obtain these communal resources [22]. The active role assigned to the various agents shows that successful transition of former child soldiers requires a network of close, informal support systems and professional support structures [1, 42]. Regarding the informal support initiatives in the community, the diversity of supports and the considerable contribution to various domains points to “community resilience”. The role fulfilled by formal support systems in addition to the organic resilience of communities indicates that wider ecological levels are also rich reservoirs of resources that can be invested in service of war-affected individuals and communities. Consequently, the term “ecological resilience” is often preferred [43]. 

The high similarity of items across informal and formal agents indicates that all agents are supposed to work toward similar goals and largely must support the same resource domains. This recommends collaborative initiatives, whereby local informal support systems within the community report to formal agents about the resources at their disposal and about their ongoing initiatives to deliver the required support. Formal support systems may in turn strengthen the local capacities by revitalizing, augmenting or formalizing the support offered by communities [19, 20, 35, 40]. Such community-based initiatives in support of former child soldiers may create an environment that fosters resource engagement and exchange, and eventually resilience of former child soldiers [44]. With regard to the role to fulfill by formal support systems, this implies that the locus of support should be communal rather than solely individual, and that their interventions should target affected communities [12]. The orientation of support to individual human resources and collective social-cultural resources indicates that specific interventions for formerly recruited youth should not be singled out, yet be integrated into wider support systems. Additionally, former child soldiers' request for support on various resource domains indicates that they do not necessarily need a singular nor an utmost specialized kind of support. This implies that supports for former child soldiers should be part of a comprehensive, multilevel initiative that operates on the individual, social and cultural dimensions of life [1, 40, 41, 45]. 

These findings should be interpreted in the light of the methodological limitations of this study. To begin with, when asked to share perspectives on desirable support, people are likely to favor direct needs satisfaction to compensate direct resource loss [21, 22]. As a consequence, it is possible that certain types of support have been underestimated and therefore were not reported, yet they could make a valuable contribution in a rather indirect manner or in the longer term. Thus, the motivation for direct needs satisfaction might have biased the participants' responses. Moreover, the hypothetical question on what might be done to support formerly recruited young people may inadvertently have raised the expectation of actually acquiring the requested support. This implies the possibility that the participants exaggerated their recommendations for informal and formal support systems, while neglecting their extant resources in hopes of emphasizing the much needed additional support [46]. Moreover, the difference in written and oral administration of the questionnaire might have generated an inadvertent bias in the data collection. Another constraint is related to the limited perspectives included in this study, given that only the non-recruited age mates were heard as representatives of the different informal and formal agents. In order to evaluate whether former child soldiers' requests for support can rely on social backing and are feasible, a thorough examination of the perspectives and resources of all different agents should occur. This could facilitate a better estimation of the extent to which formerly recruited young people's needs can be satisfied or are frustrated by environmental support systems, which is an important determinant of well-being [47].

## Figures and Tables

**Table 1:** Proportion (*n* (%)) of formerly recruited (*n* = 330) and non-recruited (*n* = 677) youth's answers per resource domain and agent.

	Themselves			Family			Friends			Organizations			Community			Government		
	Recruited *n* (%)	Non-recruited *n* (%)	*χ* ^2^	Recruited *n* (%)	Non-recruited *n* (%)	*χ* ^2^	Recruited *n* (%)	Non-recruited *n* (%)	*χ* ^2^	Recruited *n* (%)	Non-recruited *n* (%)	*χ* ^2^	Recruited *n* (%)	Non-recruited *n* (%)	*χ* ^2^	Recruited *n* (%)	Non-recruited *n* (%)	*χ* ^2^
*Human capacities *	**243** **(73.64)**	**539** **(79.62)**	**4.57**	**288** **(87.27)**	**571** **(84.34)**	**1.52**	**277 ** **(83.94)**	**558** **(86.85)**	**0.36**	**237** **(71.82)**	**420** **(62.04)**	9.36**	**228 ** **(69.09)**	**486 ** **(71.79)**	**0.78**	**184** **(55.76)**	**434** **(64.11)**	**6.52**
Mental health	97 (29.39)	249 (36.78)	5.37	52 (15.76)	163 (24.08)	9.14**	105 (31.82)	241 (35.60)	1.41	104 (31.52)	205 (30.28)	0.16	44 (13.33)	78 (11.52)	0.68	21 (6.36)	74 (10.93)	5.42
Physical health	7 (2.12)	15 (2.22)	0.01	96 (29.09)	173 (25.55)	1.42	22 (6.67)	58 (8.57)	1.10	21 (6.36)	30 (4.43)	1.72	57 (19.27)	147 (21.71)	2.71	35 (10.61)	74 (10.93)	0.02
Knowledge & skills	133 (40.30)	284 (41.95)	0.25	198 (60.00)	398 (58.79)	0.14	168 (50.91)	319 (47.12)	1.28	86 (26.06)	138 (20.38)	4.13	130 (39.39)	250 (36.93)	0.57	97 (29.39)	188 (27.77)	0.29
Livelihoods	69 (20.91)	111 (16.64)	3.08	143 (43.33)	271 (40.03)	1.00	76 (23.03)	157 (23.19)	0.00	131 (39.70)	225 (33.23)	4.05	110 (33.33)	263 (38.85)	2.89	81 (24.55)	223 (32.94)	7.42**
Personal values	32 (9.70)	68 (10.04)	0.30	30 (9.09)	34 (5.02)	6.17	23 (6.97)	44 (6.50)	0.08	17 (5.15)	32 (4.73)	0.09	8 (2.42)	22 (3.25)	0.52	0 (0.00)	2 (0.30)	0.98

*Social ecology *	**116 (35.15)**	**249 ** **(36.78)**	**0.26**	**154** ** (46.67)**	**344 ** **(50.81)**	**1.53**	**179** ** (54.24)**	**413** **(61.00)**	**4.19**	**227** ** (68.79)**	**424 ** **(62.63)**	**3.68**	**202 ** **(61.21)**	**402 ** **(59.38)**	**0.31**	**252 ** **(76.36)**	**483 ** **(71.34)**	**2.84**
Social connectedness	54 (16.36)	151 (22.30)	4.83	40 (12.12)	97 (14.33)	0.92	109 (33.03)	262 (38.70)	3.07	28 (8.48)	57 (8.42)	0.00	53 (16.06)	144 (21.27)	3.83	35 (10.61)	54 (7.98)	1.90
Social support	56 (16.97)	79 (11.67)	5.37	115 (34.85)	215 (31.76)	0.96	91 (27.58)	218 (32.20)	2.23	74 (22.42)	89 (13.15)	14.08***	111 (33.64)	175 (25.85)	6.62	44 (13.33)	84 (12.41)	0.17
Social service & infrastructure	25 (7.58)	41 (6.06)	0.84	15 (4.55)	61 (9.01)	6.34	10 (3.03)	7 (1.03)	5.33	164 (49.70)	324 (47.86)	0.30	59 (17.88)	126 (18.61)	0.08	175 (53.03)	299 (44.17)	7.00**
Safety	0 (0.00)	1 (0.15)	0.49	8 (2.42)	24 (3.55)	0.91	5 (1.52)	25 (3.69)	3.64	17 (5.15)	25 (3.69)	1.18	21 (6.36)	36 (5.32)	0.46	25 (7.58)	38 (5.61)	1.46
Law & order	7 (2.12)	13 (1.92)	0.05	1 (0.31)	1 (0.15)	0.27	0 (0.00)	5 (0.74)	2.45	7 (2.12)	26 (3.84)	2.07	7 (2.12)	16 (2.36)	0.06	88 (26.67)	177 (26.14)	0.03

*Culture & values *	**127 (38.48)**	**272 ** **(40.18)**	**0.27**	**81** ** (24.55)**	**176** ** (26.00)**	**0.25**	**103** **(31.21)**	**201** **(29.69)**	**0.24**	**33 ** **(10.00)**	**65** **(9.60)**	**0.04**	**108 ** **(32.72)**	**160** **(23.63)**	9.39**	**62** **(18.79)**	**87** **(12.85)**	**6.20**
Cultural practices	38 (11.51)	67 (9.90)	0.62	9 (2.73)	16 (2.36)	0.12	28 (8.48)	59 (8.71)	0.02	1 (0.30)	0 (0.00)	2.05	37 (11.21)	36 (5.32)	11.47***	24 (7.27)	34 (5.02)	2.07
Religious beliefs	99 (30.00)	226 (33.38)	1.16	21 (6.36)	64 (9.45)	2.74	35 (10.61)	91 (13.44)	1.63	4 (1.21)	3 (0.44)	1.90	12 (2.64)	23 (3.40)	0.04	3 (0.91)	12 (1.77)	1.13
Human rights	5 (1.52)	5 (0.74)	1.36	54 (16.36)	104 (15.36)	0.17	52 (15.76)	68 (10.04)	6.90**	29 (8.79)	63 (9.31)	0.07	69 (20.91)	111 (16.40)	3.08	42 (12.73)	42 (6.20)	12.35***

*Periphery *	**1** **(0.30)**	**0** **(0.00)**	**2.05**	**0** **(0.00)**	**0** **(0.00)**	—	**0** **(0.00)**	**0** **(0.00)**	—	**23** **(6.97)**	**31** **(4.60)**	**2.50**	**2** **(0.61)**	**2** **(0.30)**	**0.54**	**35** **(10.60)**	**69** **(10.19)**	**0.00**
Economic climate	1 (0.30)	0 (0.00)	2.05	0 (0.00)	0 (0.00)	—	0 (0.00)	0 (0.00)	—	0 (0.00)	0 (0.00)	—	2 (0.61)	1 (0.15)	1.57	12 (3.64)	12 (1.77)	3.31
Political climate	0 (0.00)	0 (0.00)	—	0 (0.00)	0 (0.00)	—	0 (0.00)	0 (0.00)	—	23 (6.97)	31 (4.60)	2.50	0 (0.00)	1 (0.15)	0.49	23 (6.97)	59 (8.71)	0.90

*Nothing *	**2** **(0.60)**	**14** **(2.07)**	**0.13**	**0** **(0.00)**	**2** **(0.30)**	**0.98**	**0** **(0.00)**	**2** **(0.30)**	**0.98**	**1** **(0.30)**	**0** **(0.00)**	**2.05**	**1** **(0.30)**	**0** **(0.00)**	**2.05**	**1** **(0.30)**	**0** **(0.00)**	**2.05**

**P ≤ .01; ***P ≤ .001.

**Table 2:** Formerly recruited youth's most frequently reported items for each agent and resource domain.

	Human Capital (*n*, %)	Social ecology (*n*, %)	Culture and values (*n*, %)	Periphery (*n*, %)
	to do agricultural activities (KS) (49, 14.85)	to take their problem to organizations and ask for support (SSI) (18, 5.46)	to pray to God (RB) (57, 17.27)	to work hard for the development of the country (EC) (1, 0.30)
Themselves	to start or continue studying (KS) (42, 12.73)	to organize themselves in a self-help group (SS) (13, 3.94)	to always put God first (RB) (25, 7.58)	
	to forget about the past (MH) (32, 9.70)	to join a youth club or organization (SC) (11, 3.33)	to behave respectful to others (CP) (15, 4.55)	

	to support them in education and training (KS) (149, 45.15)	to give them parental care (SS) (50, 15.15)	to avoid segregating them from the other children in the family (HR) (22, 6.67)	/
Family	to provide them basic requirements (L) (76, 23.03)	to show love to them (SS) (38, 11.52)	to avoid mistreating them (HR) (11, 3.33)	
	to feed them properly with balanced diet (PH) (68, 20.61)	to stay close to them (SC) (15, 4.55)	to avoid isolating them from others (HR) (7, 2.12)	

	to give them advice (KS) (99, 30.00)	to stay close to them (SC) (54, 16.36)	to avoid insulting them (HR) (25, 7.58)	/
Friends	to counsel them (MH) (30, 9.09)	to show love to them (SS) (38, 11.52)	to do storytelling with them (CP) (17, 5.15)	
	to share their properties with them (L) (29, 8.79)	to play games with them (SS) (32, 9.70)	to treat them equally to other children (HR) (11, 3.33)	

	to support them in education and training (KS) (203, 61.52)	to organize free medical care (SSI) (30, 9.09)	to advocate for these children's rights (HR) (5, 1.52)	to settle peace in the area (PC) (3, 0.91)
Organizations	to provide them basic requirements (L) (60, 18.18)	to organize care for the most vulnerable and needy (SSI) (16, 4.85)	to talk to them in a good, friendly way (HR) (1, 0.30)	to encourage and facilitate peace talks with the rebels (PC) (2, 0.61)
	to give them scholastic materials (L) (59, 17.88)	to build schools (SSI) (12, 3.64)	to promote the right for good medical care (HR) (1, 0.30)	to monitor country-level political affairs (PC) (1, 0.30)

	to support them in education and training (KS) (74, 22.42)	to care for them as their children (SS) (24, 7.27)	to treat them equally to other members of the community (HR) (29, 8.79)	to work hard for the development of the country (EC) (2, 0.61)
Community	to make sure they are fed properly (PH) (52, 15.76)	to show love to them (SS) (17, 5.15)	to introduce them to community norms (CP) (15, 4.55)	
	to give them clothes and shoes (L) (34, 10.30)	to welcome them back when they return (SC) (16, 4.85)	to avoid insulting them (HR) (12, 3.64)	

	to support them in education and training (KS) (106, 32.12)	to organize affordable or free education (SSI) (48, 14.55)	to make sure their rights are not abused (HR) (6, 1.82)	to build peace and stability in the country (PC) (27, 8.18)
Government	to provide them basic requirements (L) (42, 12.73)	to build schools (SSI) (29, 8.79)	to support them equally to other citizen (HR) (5, 1.52)	to create job opportunities (EC) (16, 4.85)
	to give them food and water (PH) (37, 11.21)	to provide free medical care (SSI) (26, 7.88)	to control if they are taken back to school (HR) (4, 1.21)	to organize peace talks with the rebels (PC) (9, 2.73)

MH: mental health; PH: physical health; KS: knowledge and skills; L: livelihoods; SC: social connectedness; SS: social support; SSI: social service and infrastructure; CP: cultural practices: RB: religious beliefs; HR: human rights; EC: economic climate; PC: political climate.
